# WGCNA identification of TLR7 as a novel diagnostic biomarker, progression and prognostic indicator, and immunotherapeutic target for stomach adenocarcinoma

**DOI:** 10.1002/cam4.3946

**Published:** 2021-05-12

**Authors:** Qihang Yuan, Qi Zhou, Jie Ren, Guan Wang, Chunlai Yin, Dong Shang, Shilin Xia

**Affiliations:** ^1^ Department of General Surgery The First Affiliated Hospital of Dalian Medical University Dalian, Liaoning China; ^2^ Clinical Laboratory of Integrative Medicine The First Affiliated Hospital of Dalian Medical University Dalian, Liaoning China; ^3^ Department of Immunology College of Basic Medical Science Dalian Medical University Dalian, Liaoning China; ^4^ Department of Oncology The First Affiliated Hospital of Dalian Medical University Dalian, Liaoning China

**Keywords:** biomarkers, immunotherapeutic targets, prognosis, stomach adenocarcinoma, weighted gene co‐expression network analysis

## Abstract

Stomach adenocarcinoma (STAD) is a malignant tumor with high histological heterogeneity. However, the potential mechanism of STAD tumorigenesis remains to be elucidated. The purpose of our research was to identify candidate genes associated with the diagnosis, progression, prognosis, and immunotherapeutic targets of STAD. Based on tumor samples from the GSE28541 dataset, weighted gene co‐expression network analysis revealed 16 modules related to STAD stage and grade. The salmon module emerged as the most relevant module (cor = 0.34), and functional enrichment analysis showed that the genes in the salmon were primarily related to major histocompatibility complex, immune response, and cell differentiation. Toll‐like receptor 7 (TLR7) was recognized as the real hub gene in the salmon module. Compared to normal stomach tissues, the transcriptional and translational levels of TLR7 were significantly elevated in STAD. Receiver operating characteristic curves verified that TLR7 displayed remarkable sensitivity and specificity for the diagnosis of STAD. The functions of TLR7 were primarily enriched in the regulation of Toll‐like receptor signaling pathway, pattern recognition receptor signaling pathway, and innate immune response. Overexpression of TLR7 tended to indicate more advanced STAD, higher degree of STAD, and poorer prognosis of STAD. In addition, TLR7 expression was positively correlated with immune cell infiltration and immune checkpoint expression. Somatic copy number alteration of TLR7 was also significantly related to immune cell infiltration. In conclusion, this study revealed the crucial role of TLR7 in STAD and provided new perspectives for the selection of biomarkers, progression and prognosis indicators, and immunotherapeutic targets for STAD.

## Graphical abstract

Combined with WGCNA analysis and data mining, TLR7 were found to be significantly associated with the progression and prognosis of STAD. TLR7 could serve as diagnostic biomarkers, prognostic indicatiors, and immunotherapeutic targets of stomach adenocarcinoma.

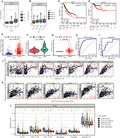

## 1 INTRODUCTION

Gastric cancer is the second leading cause of cancer‐related mortality worldwide, with an estimated 7,20,000 deaths per year.^1^ It has been well established that gastric cancer is mainly derived from the glandular epithelium, with stomach adenocarcinoma (STAD) as the most common pathology type.^2^ However, knowledge of the specific pathogenesis of STAD remains limited. It is generally accepted that various intertwined factors, including genetic susceptibility and environmental stimuli (such as cigarettes, diets, and *Helicobacter pylori* infection), contribute to morbidity and mortality in patients with STAD.^3^ Despite improvements in chemotherapy, radiotherapy, and surgery for STAD, 60% of patients with STAD are initially diagnosed with the advanced disease, thereby resulting in poor clinical outcomes.^4^, ^5^ Thus, the search for novel molecular targets has become imperative for advancing targeted therapy for STAD.

With the widespread application of high‐throughput technology, including gene chip and RNA sequencing, bioinformatics has been utilized to enable in‐depth studies on the mechanisms accounting for cancer progression. For example, some studies have adopted bioinformatics to determine the potential molecules associated with tumor progression and prognosis.^6^, ^7^ As some studies are limited in screening differentially expressed genes (DEGs) between normal and cancerous tissues,^8^, ^9^ a mechanism underlying the association between candidate genes and cancer promotion must be discovered.

In this study, we conducted weighted correlation network analysis (WGCNA) and integrated high‐connective genes into the same modules. After performing a survival analysis of all eligible genes in the selected module, the current data were found to support the prognostic role of Toll‐like receptor 7 (TLR7). Moreover, we combined data from a large number of databases to determine the ability of TLR7 to distinguish patients with STAD from healthy individuals, to validate the prognostic performance of TLR7 in STAD, and to uncover the significant contribution of TLR7 in the immune microenvironment of STAD. Overall, our findings provide novel insights into the role of TLR7, which can be exploited for novel and personalized treatment of patients with STAD.

## 2 MATERIALS AND METHODS

### 2.1 Data acquisition and preprocessing

**FIGURE 1:**
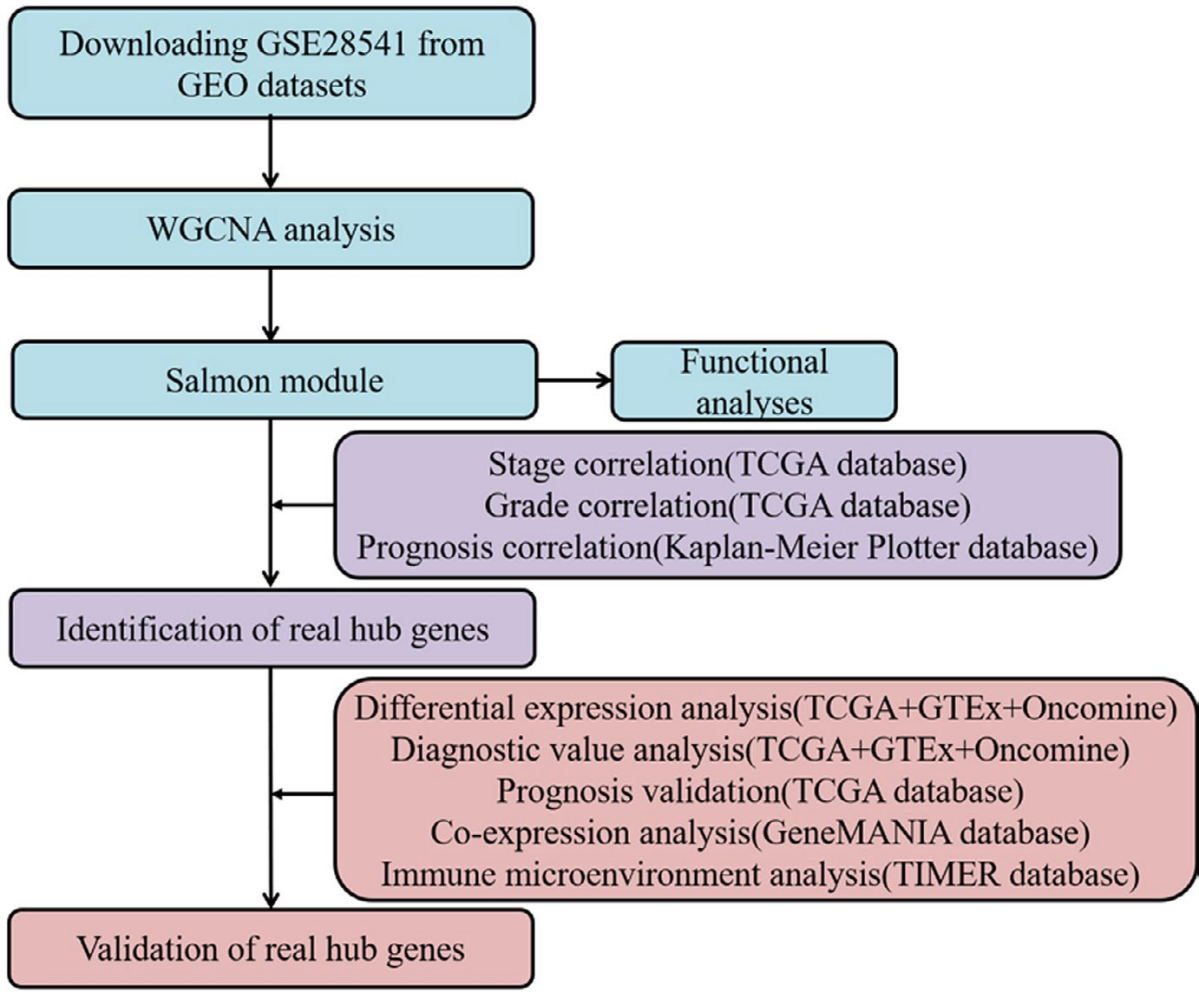
Workflow of our research

A flow diagram of the study is shown in Figure 1. We systematically searched the GEO database to identify the gastric cancer‐related datasets that were clearly diagnosed as adenocarcinoma and provided the stage and grade of each patient for further analysis. The gene chip raw data of GSE28541 provided by Sangbae et al. ^10^ were obtained from the GEO database. GSE28541, based on the GPL13376 platform (Illumina HumanWG‐6 v2.0 expression beadchip), contained 40 tumor samples with a pathologically confirmed diagnosis of STAD. In R, the robust multiple array averaging (RMA) algorithm in the affy package was implemented to preprocess the gene expression profile data. After correcting the background, quantile normalization, and probe summarization, the most variable 10,000 of 25,036 genes were preserved for WGCNA.

### 2.2 Construction of the co‐expression network

The “WGCNA” package in R was employed to establish a gene co‐expression network of the 10,000 genes.^11^ “GoodSamplesGenes” function was implemented to examine the quality of raw data. Thereafter, we obtained an adjacency matrix using Pearson's correlation analysis. We constructed a scale‐free co‐expression network by calculating the soft‐thresholding parameter β. To intensively analyze the functional modules, the adjacency matrix was converted into a topological overlap matrix (TOM), and the dissimilarity matrix between genes was calculated (dissTOM = 1‐TOM).^12^ Hierarchical clustering of dissTOM resulted in genes with similar expression clustered in the same gene module. The minimum number of module genes was set to 40.^13^ The DynamicTreeCut algorithm was applied to obtain the gene modules and the modules with high similarity were further merged.

### 2.3 Identification of the most related module and module functional annotation

Module eigengenes (MEs) and gene significance (GS) were employed to identify modules related to tumor stage and histological grade.^14^ The MEs were considered as the major element of each gene module, and ME expression was recognized on behalf of all genes in a specific module. The correlation between tumor stage, tumor grade, and MEs was derived to determine the modules associated with clinical significance. Additionally, in the linear regression analysis of clinical characteristics and gene expression profiles, GS was interpreted as the mediating *p* value of each gene (GS = lgP). The module significance (MS) was then interpreted as the average GS of all genes in a given module. The module (i.e., salmon module) with the most absolute MS was defined as the clinically significant module.

To further explore the function of the salmon module, which was obviously connected to the histologic grade of the tumor, we uploaded all genes in the salmon module into the g:Profiler website (http://www.biit.cs.ut.ee/gprofiler/gost) for functional annotation analysis and visualized the results using the Cytoscape software.^8^

### 2.4 Identification of the real hub genes in STAD

Each gene module membership (MM) was calculated in the hub module, which was used to measure the importance of each gene in the module. Genes with |GS| >0.2 and |MM| >0.8 were considered as candidate hub genes.^15^ Thereafter, 47 candidate hub genes were uploaded to the STRING database (https://string‐db.org/) to establish a protein‐protein interaction (PPI) network.^16^ The following filter conditions of the real hub genes were employed in our study: (ⅰ) high‐connective genes in the PPI network; (ⅱ) genes significantly associated with STAD stage and grade; and (ⅲ) genes significantly associated with the prognosis of STAD through analysis of the Kaplan–Meier Plotter database. Thus, only genes significantly related to both the progression and prognosis of patients with STAD were considered as the real hub genes. Of note, the minimum interaction score of these genes was >0.4, and genes with node connectivity >10 (total edges/total nodes) were considered as the hub nodes in the PPI network.^9^

The Cancer Genome Atlas (TCGA, https://cancergenome.nih.gov/) database, which has 325 STAD samples with complete clinical information, was used to identify the hub genes in the PPI network that were significantly associated with STAD stage and grade. The Kaplan–Meier Plotter database (http://kmplot.com/analysis/) is an online prognostic analysis tool that integrates the Gene Expression Omnibus (GEO), European Genome‐Phenome Archive (EGA), and TCGA databases. The Kaplan–Meier Plotter database was thus employed to assess the prognostic values of the hub genes closely related to tumor stage and grade; the following parameters were selected: “Auto select best cutoff,” “overall survival (OS),” “relapse‐free survival (RFS),” and “Stomach adenocarcinoma (n = 375)”. The hub genes that were significantly related to both the OS and RFS of STAD were preserved. Ultimately, the genes significantly associated with stage, grade, and prognosis of STAD were identified as hub genes.

### 2.5 Intensive analysis of the real hub genes through a series of databases

#### 2.5.1 Transcriptional level analysis of real hub genes in STAD and normal stomach tissues

To investigate the differences in the transcriptional levels of hub genes between STAD and normal stomach tissues and to explore the potential diagnostic value of real hub genes in STAD, the mRNA expression profiles of real hub genes were downloaded from the TCGA and Genotype‐Tissue Expression project (GTEx, https://www.gtexportal.org/home/). All data from TCGA and GTEx were converted into log_2_(fpkm+1) form for further analysis. The “sva” package in R was utilized to batch normalize the gene expression profile data from different databases. The “stat_compare_means” function in R was applied to analyze and screen the differential expression of real hub genes in STAD and normal stomach samples. The “pROC” package in R was implemented to analyze the diagnostic values of real hub genes in STAD. The Oncomine database (https://www.oncomine.org) was used to verify the different expression and diagnostic values of real hub genes; the following parameters of the real hub genes were selected: “Gene: real hub genes,” “Analysis Type: Gastric Cancer versus Normal Analysis,” “Data Type: mRNA,” and “THRESHOLD BY: *P*‐VALUE = 0.05, FOLD CHANGE = 2, GENE RANK = ALL”.

#### 2.5.2 Translational level analysis of the real hub genes in STAD and normal stomach tissues

The Clinical Proteomic Tumor Analysis Consortium (CPTAC, https://cptac‐data‐portal.georgetown.edu/) database integrates genomic and proteomic data to identify and describe all proteins in tumor and normal tissues, and to identify candidate proteins that can be used as tumor biomarkers. This database was employed to explore the discrepancy in the translational level of real hub genes in STAD and normal stomach samples. After deleting samples without the expression level of the real hub genes, 108 STAD and paracancerous tissues with “unshared log_2_(Fold Change) value” were obtained for further visualization of different protein levels of real hub genes in tumor and normal tissues via R language.

#### 2.5.3 Validation of the prognostic performances of real hub genes in STAD

A total of 325 STAD samples with complete prognostic information obtained from the TCGA database were used to verify the prognostic performance of the real hub genes. To better reflect the real biological function of a gene, the “surv_cutpoint” function in R was implemented to determine the optimal critical point of risk separation. According to the best cut‐off value, all patients with STAD were stratified into high and low expression of real hub genes. Survival analysis using the Kaplan–Meier method was then implemented to validate the prognostic performance of real hub genes. Univariate and multivariate Cox regression analyses based on the expression of the real hub genes and the clinicopathological traits identified in the TCGA database were applied to justify their independent prognostic abilities.

#### 2.5.4 Co‐expression analysis and immune infiltration analysis of the real hub genes in STAD

GeneMANIA (http://www.genemania.org) ^17^ was used to identify other genes that are closely associated with real hub genes based on functional association data, including protein and genetic interactions, pathways, co‐expression, co‐localization, and protein domain similarity. TIMER (https://cistrome.shinyapps.io/timer/),^18^ a user‐friendly and reliable online tool, was applied to systematically analyze immune infiltrates across a multitude of cancers. “Gene module” was used to explore the correlation between the real hub gene level and immune cell infiltration. The “SCNA module” was used to investigate the potential association between somatic copy number alteration (SCNA) of real hub genes and six immune cell infiltration abundances in STAD. In addition, the “correlation module” was employed to analyze the correlation between the real hub genes and certain common immune checkpoints (e.g., PDCD1, CD247, PDCD1LG2, CTLA4, HAVCR2, and IDO1). All statistical analyses in our research were conducted using R (Version 4.0.1), and statistical significance was set at *p* < 0.05.

## 3 RESULTS

### 3.1 WGCNA identification of key modules

**FIGURE 2:**
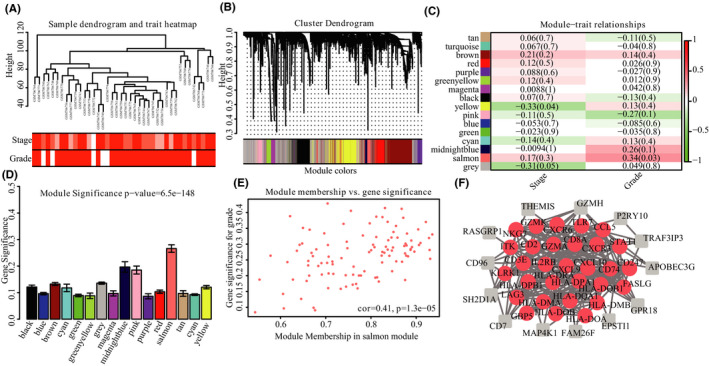
Clustering dendrogram and module identification. (A) Clustering dendrogram of 40 samples. (B) Dendrogram of all DEGs clustered based on 1‐TOM. (C) Correlation heatmap between module eigengenes and the clinical features of STAD. (D) Distribution of the average GS in the modules related to histological grades of STAD. (E) Scatter plot of module eigengenes in the salmon module. (F) PPI network of candidate hub genes derived from WGCNA (The candidate hub genes are colored in red)

**FIGURE A1:**
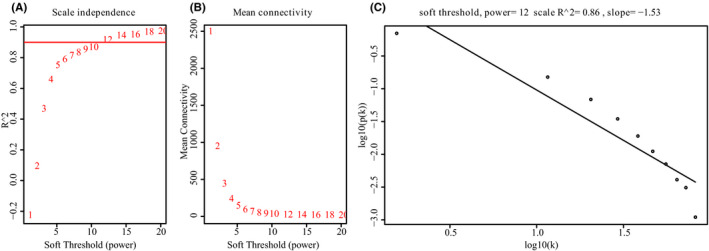
Determination of soft‐thresholding power in the WGCNA. (A) Analysis of the scale‐free fit index for various soft‐thresholding powers (β). (B) Analysis of the mean connectivity for various soft‐thresholding powers. (C) Checking the scale free topology when β = 12

The GSE28541 samples were clustered using the average linkage method and Pearson's correlation method (Figure 2A). No outlier samples were detected based on the Pearson's correlation analysis. In our research, we selected the power of β = 12 (scale‐free R^2^ = 0.86) as a soft‐thresholding parameter to determine whether the network was scale‐free (Figure A1). Based on average linkage hierarchical clustering, we identified 16 modules (Figure 2B). The salmon module was most closely related to tumor grade and was thus selected for further analysis (Figure 2C and D).

### 3.2 Biological function enrichment analysis

**FIGURE 3:**
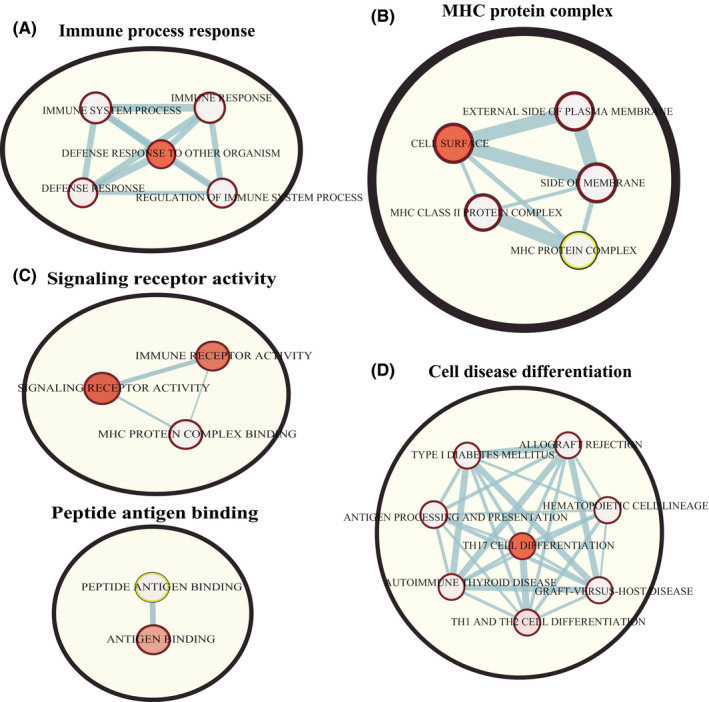
Functional analysis of all genes in the salmon module. (A) BP, (B) CC, (C) MF, and (D) KEGG

The functions of all genes in the salmon module were divided into three groups: biological process (BP), cellular component (CC), and molecular function (MF). The genes in the BP group were primarily enriched in immune response (Figure 3A); the genes in the CC group were mainly enriched in the major histocompatibility complex (MHC) protein complex (Figure 3B); and the genes in the MF group were significantly enriched in signaling receptor activity and peptide antigen binding (Figure 3C). Based on Kyoto Encyclopedia of Genes and Genomes (KEGG) pathway analysis, these candidate hub genes were primarily involved in cell differentiation (Figure 3D).

### 3.3 Identification of the real hub genes

**FIGURE 4:**
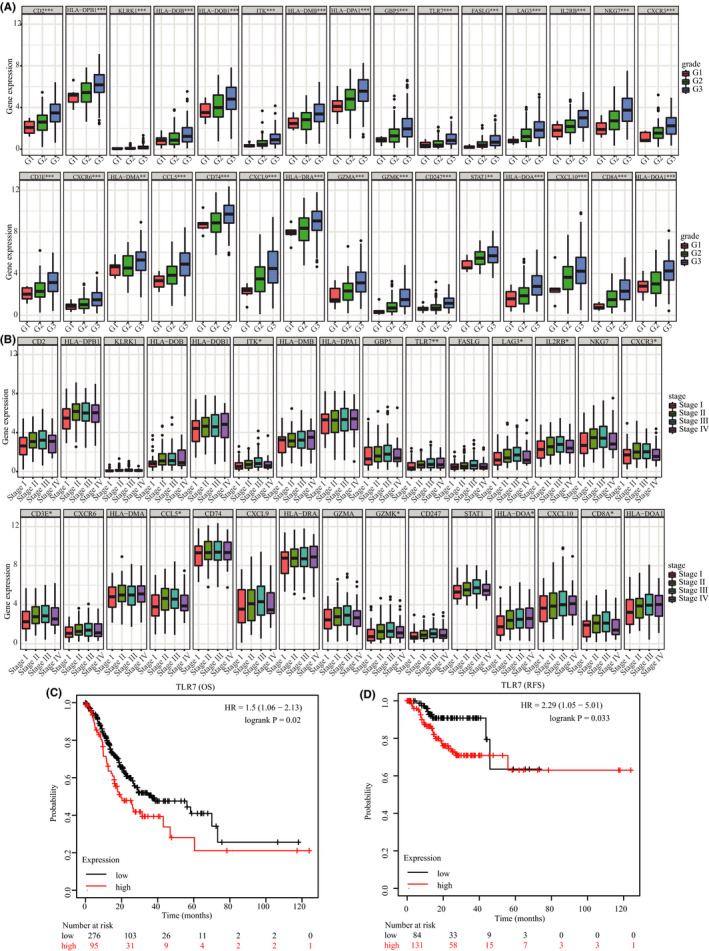
Identification of the real hub genes associated with the progression and prognosis of SATD. (A) Identification of genes significantly associated with STAD grade. (B) Identification of genes significantly associated with STAD stage. (C) Identification of genes significantly associated with the OS and RFS of patients with STAD

We identified 47 high‐connective genes in the salmon module as hub genes based on predefined criteria (Figure 2E). Thereafter, we conducted a PPI network analysis by uploading these 47 hub genes into the STRING database. Under the threshold of node connectivity >10 and a minimum interaction score >0.4, 30 hub nodes were identified (Figure 2F). Subsequently, 325 patients with STAD with complete clinical information were selected from the TCGA database to determine the association between these 30 hub genes and tumor stage and grade. The results showed that all 30 hub genes were significantly associated with tumor grade. Furthermore, 10 hub genes (i.e., ITK, TLR7, LAG3, IL2RB, CXCR3, CD3E, CCL5, GZMK, HLA‐DOA, and CD8A) were found to be significantly associated with tumor stage (Figures 4A and B). The 10 genes, closely associated with tumor stage and grade, were selected for further survival analysis using the Kaplan–Meier plotter database. Based on the results, only TLR7 was significantly associated with the OS and RFS of STAD (Figure 4C and D). Ultimately, TLR7, which is significantly related to tumor progression and prognosis, emerged as the hub gene in STAD.

### 3.4 Expression pattern analysis and diagnostic value evaluation of TLR7 in STAD

**FIGURE 5:**
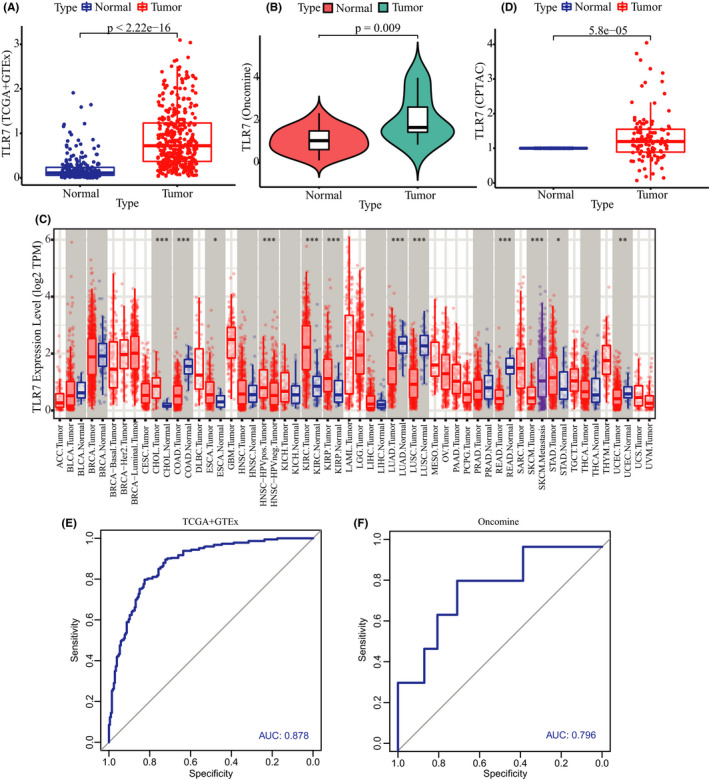
Different expression analysis of TLR7 in STAD and normal stomach tissues. (A) mRNA levels of TLR7 in STAD and normal stomach tissues based on the TCGA and GTEx databases. (B) mRNA levels of TLR7 in STAD and normal stomach tissues based on the Oncomine database. (C) Pan‐cancer analysis of TLR7 at the mRNA level based on the TIMER database. (D) Protein levels of TLR7 in STAD and normal stomach tissues based on the CPTAC database. (E) ROC curve of TLR7 based on the TCGA and GTEx databases. (F) ROC curve of TLR7 based on the Oncomine database

We evaluated the mRNA levels of TLR7 in STAD and normal stomach tissues using the gene expression profiles of TCGA and GTEx. The transcriptional level of TLR7 in STAD samples was significantly higher than that in normal stomach tissues (*p* < 0.0001) (Figure 5A). We also compared the mRNA levels of TLR7 between tumor and normal tissues using the Oncomine database (Figure 5B). Similarly, the results reached a similar conclusion (Figure 5B). Pan‐cancer analysis based on the TIMER database revealed a significantly different mRNA expression of TLR7 in many cancers, including STAD (Figure 5C). In addition, the translational level of TLR7 was found to be significantly higher in STAD tissues than in paracancerous tissues through an analysis of the CPTAC database (Figure 5D).

To estimate the diagnostic values of TLR7 in STAD, receiver operating characteristic (ROC) curves were plotted using the gene expression profiles of the TCGA, GTEx, and Oncomine databases. The area under the curve (AUC) of TLR7 was 0.878 based on the TCGA and GTEx databases and 0.796 based on the Oncomine database. Such finding indicates that TLR7 had high specificity and sensitivity for the diagnosis of STAD (Figure 5E and F).

### 3.5 Validation of the prognostic value of TLR7 in STAD

**FIGURE 6:**
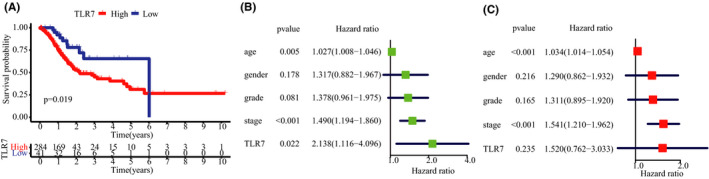
Prognostic value validation and independent prognostic analysis of TLR7 in STAD. (A) Validation of the prognostic value of TLR7 in STAD based on the TCGA database. (B) Univariate and (C) Multivariate Cox regression analyses of TLR7 in STAD

To verify the prognostic performance of TLR7 in STAD, 325 samples with corresponding survival data from TCGA were then stratified into high expression and low expression according to the best cut‐off value computed by the “surv_cutpoint” function in R. According to the survival curves depicted in Figure 6A, the high expression subgroup showed a poorer OS rate than the low‐expression subgroup. To confirm whether TLR7 acted to predict prognosis independently, univariate and multivariate Cox regression analyses were performed (Figure 6B and C). However, the results do not support the hypothesis that TLR7 is an independent prognostic factor.

### 3.6 Co‐expression analysis and immune infiltration analysis of TLR7 in STAD

**FIGURE 7:**
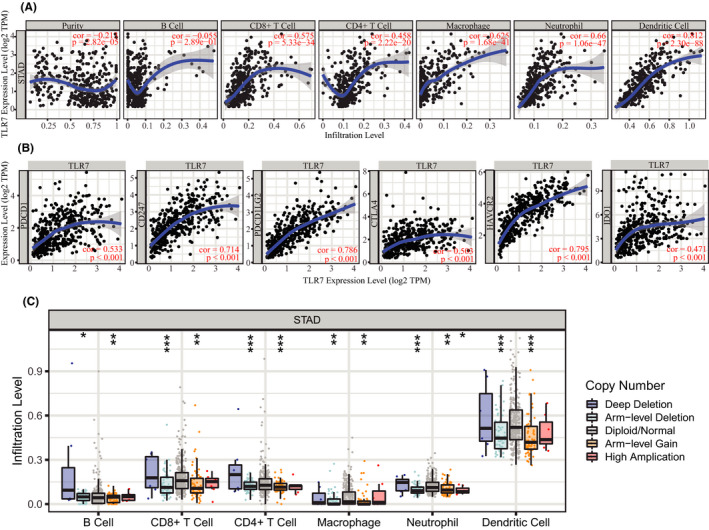
The immunoregulatory effects of TLR7 on the tumor microenvironment of STAD. (A) The relationship between TLR7 expression and the infiltration abundance of six common immunocytes in STAD. (B) The relationship between TLR7 expression and the expression of six common immune checkpoints in STAD. (C) The relationship between somatic copy number alteration of TLR7 and the infiltration abundance of six common immunocytes

**FIGURE A2:**
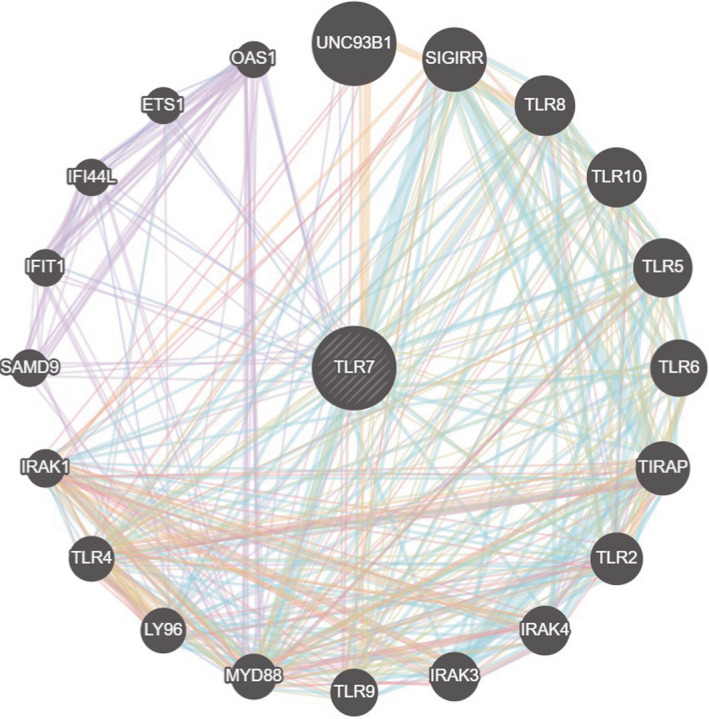
Co‐expression and interaction analyses of TLR7

GeneMANIA revealed the 20 genes most closely related to TLR7. GeneMANIA also revealed that the functions of TLR7 were primarily related to the Toll‐like receptor signaling pathway, pattern recognition receptor signaling pathway, and innate immune response‐activating signal transduction (Figure A2). In addition, the TIMER database was used to explore the correlation between TLR7 and local immune infiltration abundance in tumors. There was a significant positive correlation between TLR7 expression and the infiltration of CD8+T cells, CD4+T cells, macrophages, neutrophils, and dendritic cells (cor > 0.45, *p* < 0.001) (Figure 7A). This finding suggested that the proportion of immune cell infiltration increased with an increase in TLR7 expression. TLR7 was also found to be positively related to certain well‐recognized immune checkpoints (e.g., PDCD1, CD247, PDCD1LG2, CTLA4, HAVCR2, and IDO1) (r > 0.45, *p* < 0.001), which revealed that TLR7 might play a potential role in the response to immunotherapy in STAD (Figure 7B). In addition, different SCNAs of TLR7 were closely related to the immune infiltration of six leukocytes, indicating its regulatory role in the STAD immune microenvironment (Figure 7C).

## 4 DISCUSSION

STAD, a common alimentary tract malignancy, is associated with high morbidity and mortality. Although, progress has been made in the treatment of STAD in recent decades, the prognosis of patients remains poor. Furthermore, the pathophysiological mechanisms underlying STAD remain unclear. With the rapid development of gene chip and sequencing technology, bioinformatics analysis is playing an important role in the medical field. An increasing number of biomarkers, prognostic indicators, and immunotherapeutic targets has emerged as pivotal contributors to various malignancies.

In 2016, Yepes et al. provided a novel characterization of the miRNA expression in STAD and identified the hub miRNAs closely related to the tumoral phenotype via WGCNA.^19^ In 2018, Zhang et al. integrated gene expression profiles and corresponding clinical information of STAD from TCGA via WGCNA and revealed the prognostic role of sorting nexin 10 in STAD.^20^ In 2021, Lu uncovered the homogeneous tumorigenicity of the alimentary canal, and the SERPINE1/hsa‐mir‐145/SNHG1 axis was identified as a potential prognostic indicator and the therapeutic target of alimentary canal malignancies via WGCNA using the TCGA database.^21^ Compared to multiple analyses of the TCGA STAD cohort via WGCNA, there is an absence of the GEO STAD cohort for WGCNA. In this study, we selected GEO datasets with clinical features, including the STAD stage and grade. GSE28541 and GSE17154 were identified, which had 40 and 23 STAD samples, respectively. Given that distinct datasets in GEO resources may provide valuable complements for TCGA, GSE28541 and GSE17154 were employed to evaluate hub genes for the progression and prognosis of STAD. Of note, because a GPL platform corresponding to GSE17154 does not provide the corresponding gene bank or gene symbol, it is impossible to convert the gene ID into a well‐known gene name. Accordingly, we selected GSE28541 for our study.

In this study, WGCNA was first implemented to identify the salmon module that was most closely related to tumor grade. Functional enrichment analysis revealed that the genes in the salmon module were primarily related to the MHC protein complex, immune response, antigen‐receptor binding, and cell differentiation. A total of 30 candidate genes were identified to establish the co‐expression network and PPI network. Ten of the 30 hub genes were significantly associated with tumor stage and grade. After performing survival analysis of these 10 hub genes, only TLR7 was identified to be significantly associated with the OS and RFS of STAD and was thus considered as the real hub gene.

TLR7, a member of the Toll‐like receptor family, recognizes ligands from pathogens and dying cells, and plays an important role in the immune response through pathogen‐associated molecular patterns and damage‐associated molecular patterns.^22^ TLR7 is generally accepted to play a pivotal role in the tumor microenvironment. Research on TLR7 not only emphasizes its immunosurveillance role through the activation of innate and adaptive immune effectors, but also its promotion effect on tumor progression.^23^, ^24^ Marion et al. showed a higher expression of TLR7 in pancreatic cancer (PC) than in chronic pancreatitis, with stage‐dependent upregulation.^25^ Moreover, the activation of TLR7 facilitates PC cell proliferation and induces resistance to chemotherapy through the upregulation of NF‐κB and COX‐2 expression.^25^, ^26^, ^27^ TLR7 was also reported to be tightly associated with the prognosis of non‐small cell lung cancer by facilitating tumor progression and reducing chemosensitivity.^28^ In 2019, Diakowska et al. revealed that both the mRNA and protein expression levels of TLR7 were significantly higher in patients with gastro‐esophageal junction adenocarcinoma than in healthy individuals.^29^ At the same time, Kasurinen et al. unexpectedly found that the expression of TLR7 was significantly related to STAD stage (*p* = 0.03).^30^ Similarly, our results revealed that both the transcriptional and translational levels of TLR7 were significantly elevated in patients with STAD compared to healthy controls. Meanwhile, the expression of TLR7 was significantly associated with STAD stage and grade. The ROC curve for TLR7 displayed remarkable sensitivity and specificity for STAD. In addition, an elevated expression of TLR7 tended to predict a worse prognosis in patients with STAD. Although, our results showed that TLR7 was not an independent prognostic indicator, there is no doubt that TLR is indeed closely associated with STAD prognosis and could serve as a diagnostic biomarker and disease progression‐related predictive indicator.

Owing to an in‐depth understanding of tumor immunity, immunotherapy, especially immune checkpoint blockade (ICB), has broad application prospects in the tumor field. It has been generally accepted that ICB might provide novel insights into the treatment of advanced gastric cancers.^31^ Several studies have reported that TLR7 combined with ICB has the potential to enhance the survival of many patients with cancer. For instance, irreversible electroporation combined with a TLR7 agonist and PDCD1 blockade improved the curative effect of PC by stimulating innate and adaptive immune responses.^32^ TLR7 agonists also act to promote T lymphocyte migration into the local tissue of colon cancer. Further, when combined with PDCD1 and CTLA4 inhibitors, the infiltration of immune cells is more significantly increased.^33^ Mariola et al. suggested that TLR7 has the potential to induce CD4+T cells and CD8+T cell infiltration into the tumor microenvironment.^34^ Overall, previous studies have shown that TLR7 indeed promotes immune cell infiltration, and TLR7 combined with ICB could markedly improve the clinical outcomes of many patients with cancer. However, the interaction between TLR7 and immune checkpoints has rarely been reported.

Similarly, our results showed that TLR7 not only participated in the progression and prognosis of STAD, but also played a pivotal role in the immune microenvironment of STAD. The infiltration of T lymphocytes, macrophages, neutrophils, and dendritic cells was positively correlated with TLR7 expression. In addition, we found that the expression of TLR7 was positively associated with six common immune checkpoint expression. It has been well established that immune checkpoints negatively regulate the anti‐tumor immunity of the body, and TLR7 might attenuate the anti‐tumor immune response by increasing the expression of immune checkpoints. Dysregulation of the tumor immune microenvironment affected by TLR7 overexpression might be responsible for the poor prognosis of STAD. Altogether, these findings enhance our understanding of these checkpoints in cancer therapy and provide novel insights into immune checkpoint‐targeting therapeutic strategies for STAD.

Our study had some limitations. Our eligibility criteria for the GEO datasets were: (ⅰ) the pathological type must be clearly diagnosed as adenocarcinoma and (ⅱ) the stage and grade information of each patient must be available. Hence, although, there was a collection of gastric cancer‐related datasets in the GEO database, most of the datasets were not eligible for our study, which led to a relatively small number of patients with STAD being used for WGCNA. In addition, this study is a retrospective study based on bioinformatics analysis. Therefore, more solid experiments and well‐designed prospective studies are warranted to verify our findings and highlight the crucial role of TLR7 in the occurrence and development of STAD.

In conclusion, after constructing the co‐expression network and intensively analyzing multiple databases, one real hub gene (i.e., TLR7) that could serve as a biomarker for the diagnosis, progression, and prognosis of STAD was identified layer by layer, thereby providing new insights into the pathogenesis of STAD. More importantly, TLR7 is expected to be a novel therapeutic target for STAD.

## 5 CONFICT OF INTEREST

All authors declare that there is no conflict of interest.

## AUTHOR CONTRIBUTIONS

The authors alone are responsible for the content and writing of the paper. Qihang Yuan contributed to the design of the study, collection, interpretation of data, drafting, and revising the manuscript. Qi Zhou and Jie Ren participated in the design of the study, collection of data and drafting the manuscript. Guan Wang and Chunlai Yin were responsible for the collection and interpretation of data. Shilin Xia and Prof. Dong Shang conceived the study and reviewed/edited the manuscript.

## ETHICAL APPROVAL STATEMENT

Not applied.

## Data Availability

All the data are available upon request.
